# TTX-Resistant NMDA Receptor-Mediated Membrane Potential Oscillations in Neonatal Mouse Hb9 Interneurons

**DOI:** 10.1371/journal.pone.0047940

**Published:** 2012-10-18

**Authors:** Mark A. Masino, Matthew D. Abbinanti, John Eian, Ronald M. Harris-Warrick

**Affiliations:** 1 Department of Neuroscience, University of Minnesota, Minneapolis, Minnesota, United States of America; 2 Department of Neurobiology and Behavior, Cornell University, Ithaca, New York, United States of America; Georgia State University, United States of America

## Abstract

Conditional neuronal membrane potential oscillations have been identified as a potential mechanism to help support or generate rhythmogenesis in neural circuits. A genetically identified population of ventromedial interneurons, called Hb9, in the mouse spinal cord has been shown to generate TTX-resistant membrane potential oscillations in the presence of NMDA, serotonin and dopamine, but these oscillatory properties are not well characterized. Hb9 interneurons are rhythmically active during fictive locomotor-like behavior. In this study, we report that exogenous N-Methyl-D-Aspartic acid (NMDA) application is sufficient to produce membrane potential oscillations in Hb9 interneurons. In contrast, exogenous serotonin and dopamine application, alone or in combination, are not sufficient. The properties of NMDA-induced oscillations vary among the Hb9 interneuron population; their frequency and amplitude increase with increasing NMDA concentration. NMDA does not modulate the T-type calcium current (I_Ca(T)_), which is thought to be important in generating locomotor-like activity, in Hb9 neurons. These results suggest that NMDA receptor activation is sufficient for the generation of TTX-resistant NMDA-induced membrane potential oscillations in Hb9 interneurons.

## Introduction

In vertebrates, neural circuits called central pattern generators (CPGs) that produce rhythmic motor output during locomotion are located in the ventromedial region of the spinal cord [1]–[9]. Motor activity in many rhythm-generating networks is determined, in part, by the intrinsic properties of the constituent neurons [10]–[17]. These intrinsic properties are determined by the repertoire of ionic currents expressed by individual neurons [16], [18]–[20]. For example, NMDA receptors in vertebrates are activated in spinal circuits during locomotor activity [1], [21]–[25] and NMDA is known to induce TTX-resistant membrane potential oscillations in a number of neuron types in various regions of the central nervous system [26]–[36]. Although the identities of the neural components and the cellular mechanisms that participate in driving rhythmic locomotor activity in vertebrate CPGs are not well understood, conditional neuronal membrane potential oscillations have been identified as a potential mechanism to help support rhythmogenesis in neural circuits [26]–[34], [36].

A recent strategy to characterize the cellular properties that promote voltage oscillations has been to monitor the activity of identified neuronal populations from transgenic lines of mice that express fluorescent proteins under the control of specific promoter constructs, such as transcription factors [35], [37]–[44]. Using this approach, it has been shown that the Hb9 interneurons, a class of ventromedial excitatory interneurons, are rhythmically active during fictive locomotion in neonatal mice [40], [41]. Further, Hb9 interneurons appear to be conditional oscillators since they generate TTX-resistant membrane potential oscillations in the presence of NMDA, serotonin and dopamine [35], [41]. Although Hb9 interneurons are unlikely to be, by themselves, responsible for producing the locomotor rhythm in neonatal mice [35], [45], [46], their bursting properties, location, and rhythmicity during locomotor activity have led to the suggestion that these interneurons play a role in generation of the locomotor pattern [40], [41], [47]–[50]. Thus, we studied the membrane properties underlying their rhythmicity. We find that NMDA receptor activation can induce strong Hb9 oscillations, and does not do so via activation of low threshold calcium currents.

## Materials and Methods

### Animals

All procedures were approved by the Institutional Animal Care and Use Committees at the University of Minnesota and Cornell University and were in accordance with National Institutes of Health guidelines. Experiments were performed on spinal cords isolated from transgenic mice (Hb9::eGFP provided by Robert Brownstone, Dalhousie University) from post-natal day 3 (P3) to P9. Animals were euthanized by acute decapitation, as recommended by the AMVA Panel on Euthanasia.

### Spinal Cord Preparation

The spinal cord from T9-S1 was removed by laminectomy in ice-cold (4°C), oxygenated (95% O_2_/5% CO_2_) low calcium Ringer's solution (in mM: 128 NaCl, 4.7 KCl, 1.2 KH_2_PO_4_, 0.25 CaCl_2_, 1.3 MgCl_2_, 3.25 MgSO_4_, 25 NaHCO_3_, and 22 D-glucose). The meninges were removed and the cord was imbedded in 3.7% agarose (Invitrogen; UltraPure Agarose) in either HEPES Ringer's solution (in mM: 101 NaCl, 3.8 KCl, 18.7 MgCl_2_, 1.3 MgSO_4_, 1.2 KH_2_PO_4_, 1.0 CaCl_2_, 10 HEPES and 25 D-glucose; pH to 7.4 with NaOH) or sucrose solution (in mM: 188 sucrose, 25 D-glucose, 26 NaHCO_3_, 25 NaCl, 10 MgSO_4_, 1.2 NaH_2_PO_4_ and 1.9 KCl; pH to 7.4 with NaOH). The imbedded spinal section was transferred to a vibrating microtome (Leica, VT1000S or VT1200S), and transverse sections (200–300 µm) of the L1-L3 region were cut in HEPES Ringer's or sucrose solution at 0°C, transferred immediately to an incubation chamber containing pre-warmed (30°C), oxygenated (95% O_2_/5% CO_2_) normal Ringer's solution and allowed to equilibrate for at least 30 minutes before starting the experiment.

### Pharmacology

The following channel blockers were used: TTX (1 μM, to block voltage-gated sodium current), tetraethylammonium chloride (TEA-Cl, 30 mM to block voltage-gated potassium current), 4-aminopyridine, (4-AP, 4 mM, to block fast transient potassium current), CsCl (2 mM, to block hyperpolarization activated inward current). Pharmacological agents used to evoke endogenous membrane potential oscillations in slice preparations were N-Methyl-D-aspartic acid (NMDA 3–21 μM), serotonin creatinine sulfate complex (5-HT 3–21 μM) and dopamine hydrochloride (DA 15–50 μM), all obtained from Sigma-Aldrich. All agents were dissolved in normal Ringer's solution and applied at 20–22°C.

### Electrophysiological Recordings

To make standard whole-cell patch recordings, slices were transferred from the warm (∼30°C) incubation chamber to the recording chamber, and equilibrated with oxygenated (95% O2/5% CO2) normal Ringer's solution at room temperature for at least 10 minutes before starting the experiment. Cells expressing GFP were identified under epifluorescent illumination and visualized for targeted recording using infrared differential interference contrast optics (BX51WI; Olympus). The following criteria were used to validate the identity of potential Hb9::GFP spinal interneurons [41], [50]: 1) GFP-positive somata located in the ventromedial spinal cord just ventral to the central canal; 2) somata typically arranged in groups of two to three cells [42]; 3) characteristic bipolar shape along the dorsoventral axis; 4) a single projection fiber extending from each of the soma; and 5) key electrophysiological characteristics, including lack of sag potential during hyperpolarizing current steps, and marked post-inhibitory rebound with doublet-spikes [41], [45], [47].

Patch electrodes (∼8–10 M**Ω**) were pulled on a Flaming/Brown micropipette puller (P-97, Sutter Instruments) from borosilicate glass (1.5 mm OD, 0.86 mm ID, Warner Instruments). For current-clamp recordings, the patch electrodes were filled with the following intracellular solution (in mM): 138 K gluconate, 0.0001 CaCl_2_, 10 HEPES buffer, 5 Mg-ATP and 0.3 GTP-Li, adjusted to pH 7.3 with KOH. For voltage-clamp recordings of calcium currents, the patch electrodes were filled with the following intracellular solution (in mM): 100 CsCl, 30 TEA-Cl, 0.5 CaCl_2_, 1 MgCl_2_, 10 HEPES buffer, 5 Mg-ATP and 0.3 GTP-Li, 5 NaCl, 10 EGTA, adjusted to pH 7.3 with KOH.

#### Whole-cell current and voltage clamp

For current- and voltage-clamp recordings, whole-cell voltage was monitored and controlled with a MultiClamp 700B amplifier (Molecular Devices). Data were filtered at 30 kHz and digitized at 66 kHz. The recordings were accepted for data analysis if the resting membrane potential was more negative than −45 mV and the cells generated overshooting (>0 mV) spikes.

To isolate low threshold T-type calcium currents for voltage-clamp recording, we blocked Na^+^ channels with TTX (1 µM) and K^+^ channels with TEA-Cl (30 mM) and 4-AP (4 mM) added to the extracellular solution, and 100 mM CsCl and 30 mM TEA-Cl replacing K^+^ in the intracellular solution. Although apparently not present in Hb9 interneurons, hyperpolarization-activated inward (I_h_) channels were blocked with 2 mM extracellular CsCl. To allow a satisfactory block of Na^+^ and K^+^ currents, at least 5 min superfusion of extracellular blockers was performed before initiation of voltage clamp experiments. Low voltage step protocols were designed to activate T-type Ca^2+^ currents. The membrane potential was clamped to −100 mV and a step of ∼200 msec depolarizing pulses up to −30 mV were applied. During voltage-clamp recording, the access resistance was monitored continually, and the recording was discarded if the access resistance changed by more than10% during the course of the experiment. All recorded neurons were labeled with 0.1% Sulforhodamine B (Sigma) added to the patch solution, and fluorescent images were acquired with a CCD camera (C-72-CCD, Dage MTI), a frame grabber (LG3, Scion) and imaging software (ImageJ, National Institutes of Health) for morphological identification (data not shown).

Clampfit (Molecular Devices, Sunnyvale, CA) was used to measure current in voltage-clamp mode. A program written in MATLAB (Mathworks, Natick MA) was used to analyze the current clamp data. Intracellular Hb9 recordings were DC-coupled and not rectified. First, an estimate of a global mean burst frequency was determined from an autocovariance analysis of 20–30 sec of recording, where the mean burst frequency was the reciprocal of the lag-time between the central peak of the autocovariance and the first subsequent peak. Next, the voltage recording was smoothed via low-pass Butterworth filtering with a cut-off frequency of either two or four-times the estimated global mean burst frequency (zero-phase forward-backward convolution with second order Butterworth low-pass). Finally, the smoothed voltages were used to determine characteristics of individual bursts as follows.

For current-clamp recordings, the occurrence times of rhythmic bursts in the smoothed voltages were determined with an algorithm that searched for local peaks and troughs over ¼-width intervals while forcing adjacent peaks and troughs to be separated by at least ¼-width, and additionally forcing peaks and troughs to alternate. With the peaks and troughs defined, the individual “burst sections” were then defined as the interval between adjacent troughs. To determine the start of individual bursts, the burst-onset was defined as the time where the smoothed waveform rose from the first trough to 10% of the way to the next peak. Similarly, burst-termination was defined as the time where the smoothed waveform fell from the peak by 90% of the vertical distance to the next trough.

To quantify burst voltage amplitude, we averaged the deflection of membrane potential (in mV) from trough-to-peak. To quantify “burst strengths” for individual bursts (extra- and intracellular), we integrated the area between the smoothed voltage and the straight-line connection between burst start and termination points as defined above.

The analysis program was used to determine cycle period (T; time difference between successive burst peaks), cycle frequency (CF; reciprocal of cycle period (1/T)), burst duration (BD; proportion of the cycle period occupied by the burst) and burst strength (BS; defined above) for each voltage trace. The means and standard deviations for each parameter were then determined. The analysis code is available upon request.

### Statistical Analysis

Properties of NMDA-induced oscillations (cycle frequency, voltage amplitude and burst strength) were assessed by Pearson correlation (r). Means of cycle frequency and voltage amplitude at different trough potentials were analyzed by one-way ANOVA with subsequent protected *t*-test and NMDA modulation of I_Ca(T)_ was analyzed by one way repeated measures ANOVA with Systat software (SigmaPlot, version 12). Data with a *P*<0.05 were accepted as statistically significant. Means are presented ± SD.

## Results

### NMDA receptor activation is sufficient to induce membrane potential oscillations in Hb9 interneurons

In many experimental protocols, a combination of NMDA, serotonin (5-HT) and dopamine (DA) is used to evoke fictive locomotor-like activity in the isolated mouse spinal cord [6], [40], [51]–[53]. This combination is sufficient to initiate membrane potential oscillations in Hb9 spinal interneurons when spike-mediated synaptic transmission is abolished with TTX [41], [49]. To determine whether any of the compounds used to activate fictive locomotor-like activity were independently sufficient to generate rhythmic membrane potential oscillations in Hb9 spinal interneurons, we blocked spike-mediated synaptic interactions with TTX (1 μM), applied each neuroactive compound to the neuron and monitored membrane potential using whole-cell patch recordings.

**Figure 1:**
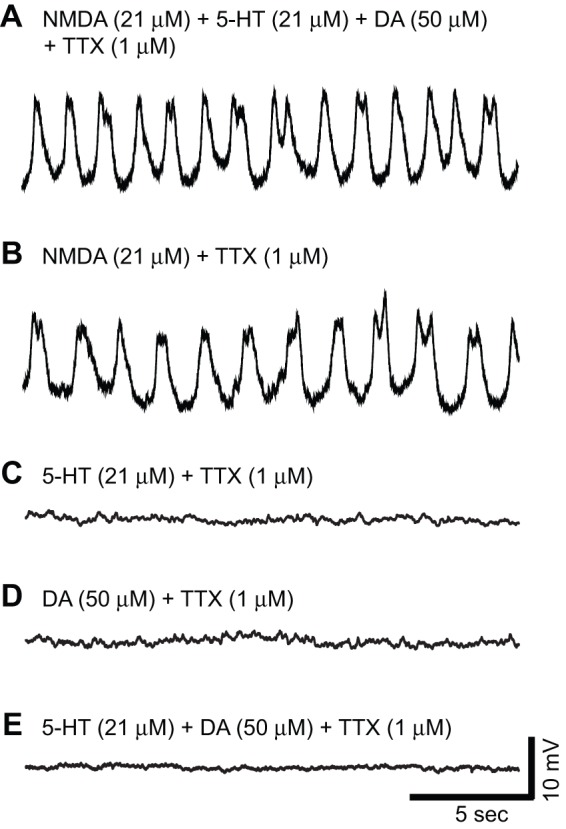
Exogenous NMDA application is sufficient to induce membrane potential oscillations in Hb9 interneurons when spike-mediated synapses are blocked with TTX. A–E, Whole-cell current-clamp recordings of membrane potential in various Hb9 interneurons under different conditions; the slow, small spikes at the tops of the oscillations are probably calcium-mediated. **A,** Membrane potential oscillations in the presence of a ‘cocktail’ of chemicals (21 μM NMDA, 21 μM 5-HT and 50 μM DA) that produces fictive locomotor-like activity in isolated whole-cord preparations. **B,** Exogenous NMDA application (21 μM) is sufficient to induce oscillations. **C–E,** Exogenous serotonin (5-HT, 21 μM) and dopamine (DA, 50 μM) application alone, or in combination, are not sufficient to induce Hb9 oscillations.

A combination (‘cocktail’) of all three neuroactive chemicals (21 μM NMDA, 21 μM 5-HT and 50 μM DA) generated membrane potential oscillations in most Hb9 interneurons (18 of 25; Fig. 1A). Bath application of NMDA alone (21 μM) produced similar oscillations in most Hb9 interneurons (40 of 61; Fig. 1B). We chose a concentration (21 μM) of NMDA shown to elicit both fictive locomotor-like activity in whole spinal cord preparations [35], [40], [51]–[53] and endogenous membrane potential oscillations in Hb9 interneurons from transverse slice preparations when applied together with 5-HT and DA [1], [6], [30]. However, neither 5-HT (21 μM; 0 of 6; Fig. 1C) nor DA (50 μM; 0 of 7; Fig. 1D) application alone, or the combination of 5-HT and DA (0 of 7; Fig. 1E), produced membrane potential oscillations. The variance of the cycle frequency did not differ between oscillations evoked by NMDA alone or the full cocktail of NMDA, 5-HT and DA (data not shown).

### Properties of NMDA-induced membrane potential oscillations

**Figure 2:**
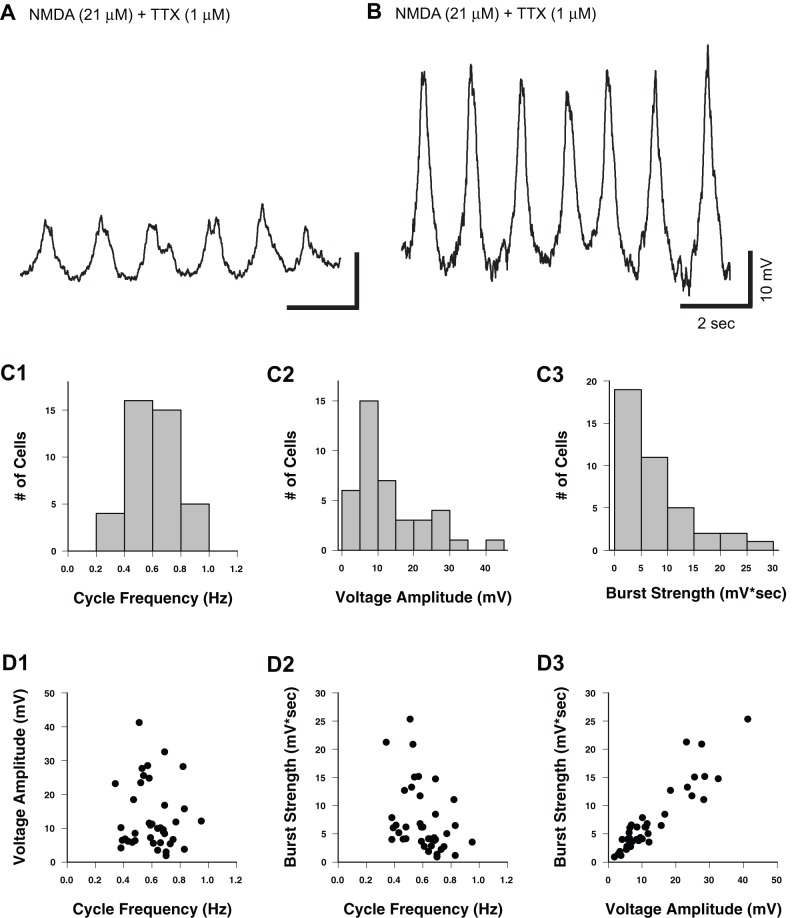
Distribution of cycle frequency and voltage amplitude for NMDA-induced membrane potential oscillations in Hb9 interneurons. A–B, Whole-cell current-clamp recordings of membrane potential from different Hb9 interneurons demonstrate variability of voltage amplitudes in NMDA induced oscillations. **C1–3,** Distribution plots of cycle frequency, voltage amplitude and burst strength. **D1–3,** There is no correlation between voltage amplitude and cycle frequency (D1; r = −0.10, p = 0.55, n = 40). However, correlations are found between burst strength and cycle frequency (D2; r = −0.39, p = 0.01, n = 40) and burst strength and voltage amplitude (D3; r = 0.92, p<0.001, n = 40).

The properties of the TTX-resistant NMDA-induced oscillations were variable between Hb9 interneurons (Fig. 2A & B). To quantify this variability, we measured cycle frequency, voltage amplitude and burst strength from a common trough potential, held at −60 mV with bias current. The cycle frequency of NMDA-induced oscillations ranged from 0.34 to 0.95 Hz (mean  = 0.61±0.15 Hz, n = 40; Fig. 2C1). The voltage amplitude of NMDA induced oscillations ranged from 1.9 to 41.2 mV (mean  = 12.8±9.5 mV, n = 40; Fig. 2C2) with 70% (28 of 40) less than 15 mV. The burst strength of NMDA-induced oscillations ranged from 0.9 to 25.3 mV*sec (mean  = 7.2±5.9 mV*sec, n = 40; Fig. 2C3) with 75% (30 of 40) less than 10 mV*sec. These values do not show a bimodal distribution, suggesting that they reflect variation of a single population of Hb9 neurons.

Next, to examine potential interactions among the oscillatory parameters in Hb9 interneurons, we tested whether the amplitude and burst strength of the NMDA-induced membrane potential oscillations varied as a function of cycle frequency. The amplitude of the NMDA-induced membrane potential oscillations was not correlated with cycle frequency (r = −0.10, p = 0.55, n = 40; Fig. 2D1). Thus, cycle frequency and voltage amplitude of the membrane potential oscillations were independent properties of the NMDA-induced activity. However, burst strength did show a significant negative correlation with cycle frequency (r = −0.39, p = 0.01, n = 40; Fig. 2D2). Thus, weak burst strength tended to occur with more rapid oscillations, as predicted by the shorter burst durations at higher cycle frequencies. As expected, burst strength was strongly correlated (r = 0.92, p<0.001, n = 40) with voltage amplitude (Fig. 2D3).

Finally, to directly test whether 5-HT and DA modified the properties of NMDA-induced Hb9 oscillations, we compared NMDA- to NMDA/5-HT/DA cocktail-induced membrane potential oscillations. Neither voltage amplitude (t = −0.1, p = 0.9) nor burst strength (t = −1.2, p = 0.3) were significantly different between the two treatments. However, the cycle frequency of cocktail-induced oscillations (mean  = 0.91±0.28 Hz, n = 18) was significantly higher than the cycle frequency of NMDA-induced oscillations (mean  = 0.61±0.15 Hz, n = 40; t = 5.5, p<0.001).

### NMDA concentration dependence of Hb9 interneuron membrane potential oscillations

**Figure 3:**
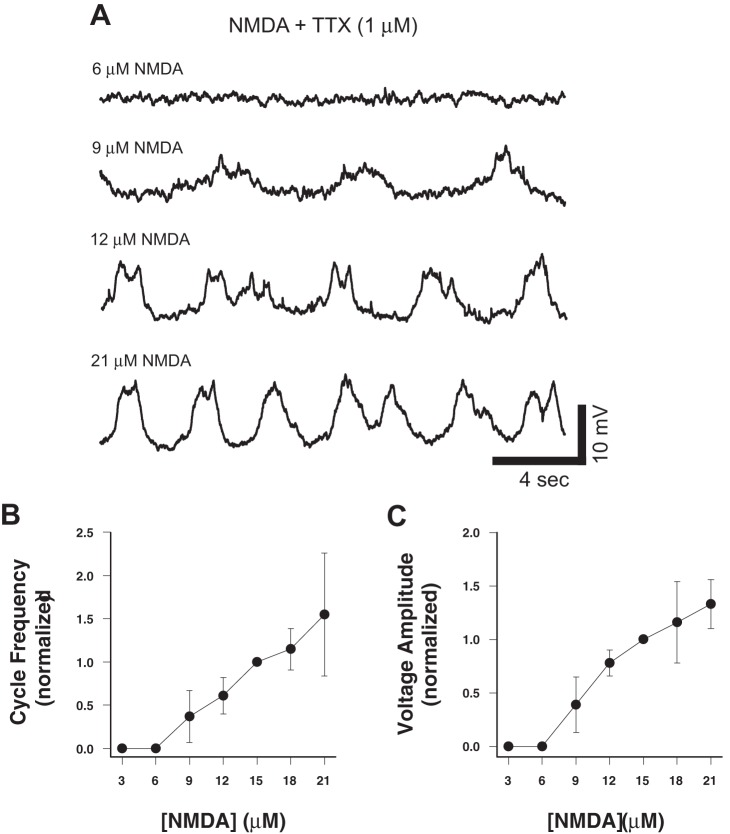
Concentration dependence of NMDA-induced membrane potential oscillations in Hb9 interneurons. A, Whole-cell current-clamp recordings of membrane potential from a single Hb9 interneuron at several NMDA concentrations. Note the lowest NMDA concentration (6 μM) does not induce oscillations. **B–C,** NMDA dose-response curves for cycle frequency show a linear relation above 6 μM (r = 0.87, p<0.001, n = 20) and voltage amplitude (r = 0.92, p<0.001, n = 20). The threshold for oscillation activation is ∼9 μM. Data are normalized to 15 μM.

To determine the NMDA concentration dependence of the Hb9 interneuron oscillations, we applied a series of NMDA concentrations (3 to 21 μM) to transverse slice preparations in the presence of TTX and monitored the Hb9 membrane potential. Lower NMDA concentrations (3 or 6 μM) did not generate membrane potential oscillations in these cells (0 of 4; Fig. 3A, top trace). Membrane potential oscillations were generated between 9 and 21 μM NMDA; the lowest effective NMDA concentration that elicited oscillations was 9 μM (3 of 4; Fig. 3B & C).

Next, we quantified the NMDA concentration-dependence of the Hb9 oscillation cycle frequency and voltage amplitude. Over a range of 9 to 21 μM NMDA, both cycle frequency (r = 0.87, p<0.001, n = 20; Fig. 3A & B) and voltage amplitude (r = 0.92, p<0.001, n = 20; Fig. 3A & C) increased roughly linearly as the NMDA concentration increased.

**Figure 4:**
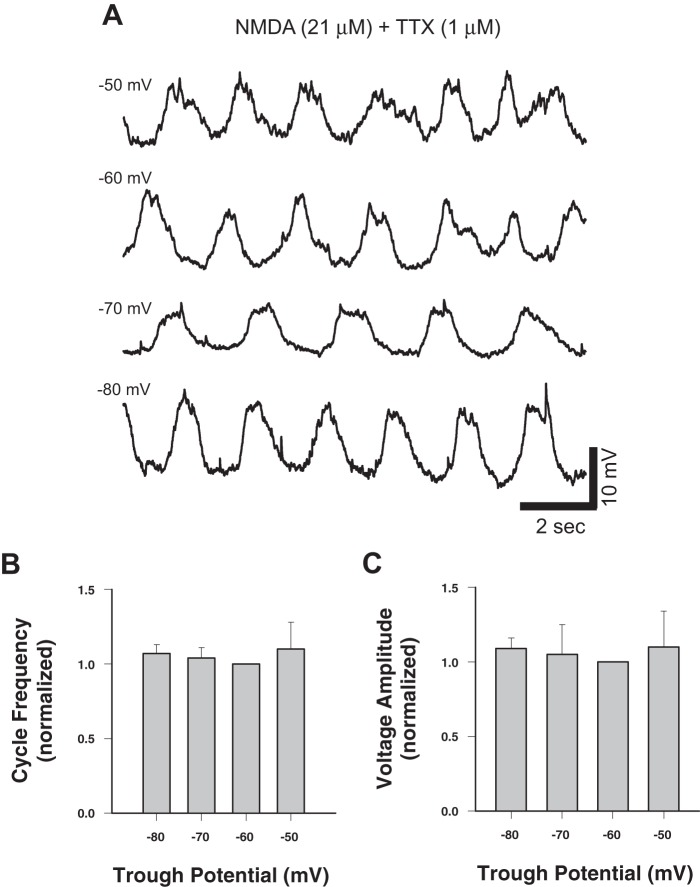
TTX-resistant NMDA-induced membrane potential oscillations in Hb9 interneurons show no voltage dependence. A, Whole-cell current-clamp recordings of membrane potential from a single Hb9 interneuron at various holding potentials. **B,** Plot of normalized cycle frequency (raw mean at −60 mV  = 0.46±0.06 Hz, range  = 0.4 to 0.5 Hz, n = 7) against holding potential. **C,** Plot of normalized voltage amplitude (raw mean at −60 mV  = 11.7±14.5 mV, range  = 4.1 to 14.5 mV, n = 7) against holding potential. Data are normalized to −60 mV.

Previous work showed that ‘cocktail’-induced membrane potential oscillations were voltage dependent in a small fraction of Hb9 interneurons [41]. To determine the voltage-dependence of cycle frequency and voltage amplitude of the NMDA-induced oscillations, we varied the trough potential of the membrane potential oscillations above and below the normal resting potential in 7 neurons (5 small amplitude (<15 mV) and 2 large amplitude (>15 mV; Fig. 4A). Neither cycle frequency (F_3,19_ = 1.09, p = 0.38; Fig. 4B) nor voltage amplitude (F_3,18_ = 0.63, p = 0.61; Fig. 4C) of the NMDA-induced oscillations was significantly affected by changing the membrane potential across a physiologically relevant range (−50 to −80 mV).

### NMDA does not potentiate the low-threshold calcium current

**Figure 5:**
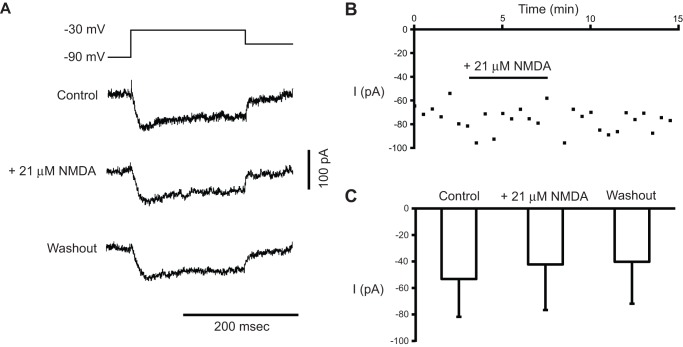
NMDA does not potentiate low-threshold calcium current in Hb9 interneurons. A, From top to bottom, current traces elicited by steps from −90 mV to −30 mV in control, 21 μM NMDA, and washout. Note the lack of effect on the current by NMDA. **B,** Time course of calcium current elicited by steps from −90 to −30 mV versus time. Time course chart and representative sweeps at top are from same experiment. **C,** Mean peak current amplitudes elicited by steps from −90 to −30 mV in control, 21 μM NMDA, and washout.

The low-threshold calcium current (I_Ca(T)_) plays a role in chemically-induced locomotor rhythmogenesis and in the generation of membrane potential oscillations in Hb9 interneurons [35], [49]. Thus, we asked whether NMDA could directly potentiate I_Ca(T)_ in Hb9 interneurons. We measured a mixture of low- and higher-threshold calcium current before, during, and after the application of 21 µM NMDA. In a series of whole-cell voltage-clamp experiments (n = 10), sodium and potassium channels were blocked and inward calcium current was measured using steps from −90 to −30 mV (4 of 10 preparations expressed I_Ca(T)_; Fig. 5A). NMDA (21 µM) did not increase the amplitude of the inward calcium current (F_2,9_ = 0.20, p = 0.83, n = 4) across a 15-minute recording window (Fig. 5B,C). This suggests that, although I_Ca(T)_ is required for NMDA-induced membrane potential oscillations [35], [49], it is not a target of NMDA modulation in Hb9 interneurons.

## Discussion

In this study, we characterized TTX-resistant NMDA-induced rhythmic membrane potential oscillations in a subpopulation of ventromedial spinal interneurons from Hb9::eGFP transgenic mice. These Hb9 interneurons are rhythmically active during chemically-induced locomotor-related activity [35], [40]–[46], [49], [50]. We focused on the effects of NMDA on the Hb9 interneurons since NMDA receptor activation can induce TTX-resistant rhythmic voltage fluctuations in several types of spinal neurons including motoneurons [26], [32]–[34], [47] and interneurons located near the central canal in neonatal rats [35], [36], [40], [41]. In addition to the Hb9 interneurons described above (Figs. 1B, 2–4), TTX-resistant NMDA-induced membrane potential oscillations have been observed in “Type 2” Hb9 interneurons [41] (unpublished data), in spinal motoneurons [54], and in two populations of genetically identified interneurons, which express engrailed-1 (En1) and single-minded homolog 1 [sim1] (Y. Zhang, personal communication). These data suggest that TTX-resistant NMDA oscillations are not exclusive to the Hb9 interneuron population and that other cell types in the ventral region of the mammalian spinal cord can produce such oscillations. Although voltage oscillations can be observed in various cell types in the spinal cord under artificial *in vitro* experimental conditions, such activity is not necessarily required, or even necessarily present, during locomotor CPG operation [6], [21], [55]. Thus, the ability of TTX-isolated neurons to produce voltage oscillations may simply reflect the presence of bi-stable membrane properties that can shape nonlinear membrane behavior in response to synaptic input during locomotor network operation [56]–[61].

In our experiments, exogenous application of NMDA, in the presence of TTX, was sufficient to generate rhythmic membrane potential oscillations in Hb9 interneurons, whereas exogenous application of 5-HT and DA, either alone (n = 6 and n = 7, respectively) or in combination (n = 7) did not evoke oscillations (Fig. 1B–E). Han et al. [62] presented evidence that exogenous application of dopamine was necessary but not sufficient to produce membrane potential oscillations in Hb9 interneurons. Our results may differ from those of Han et al. in the duration of drug application; we found that NMDA usually had to be applied for a minimum of 10 min to evoke oscillations alone in TTX.

Although NMDA alone could evoke Hb9 oscillations, our experiments cannot rule out the possibility that constitutive 5-HT and DA receptor activity may be present in our slice preparations [34], [63], [64], and that this constitutive receptor activity, in the absence of the transmitters, may play a role in generating voltage oscillations in Hb9 interneurons when NMDA alone is applied. An alternative possibility is that there is intrinsic release of 5-HT and/or DA within the preparations, which could interact with exogenous NMDA to evoke oscillations. We consider this possibility unlikely, as TTX was present in all experiments, and we used slices that had been incubated for more than one hour before the experiment started.

Although 5-HT and DA by themselves or in combination do not generate Hb9 oscillations, our results suggest that they can modify NMDA-evoked oscillatory properties [57]. NMDA- and cocktail-induced TTX-resistant oscillations were generated in similar percentages of Hb9 interneurons (66% and 72%, respectively), suggesting that 5-HT and DA did not enhance or bias the oscillatory capacity of Hb9 interneurons. Comparison of NMDA- and cocktail-induced oscillations revealed that addition of 5-HT and DA, in combination with NMDA, does not modify either voltage amplitude or burst strength compared to oscillations evoked by NMDA alone. However, the cycle frequency was significantly faster for the cocktail-induced oscillations. These results, taken together, suggest that 5-HT and DA play a modulatory role in shaping the properties of NMDA-evoked, TTX-resistant oscillations in Hb9 interneurons.

Additionally, the monoamines could alter the effective concentration of NMDA required to evoke oscillations. In our experiments applying NMDA alone, the threshold concentration of NMDA (9 μM) necessary to evoke membrane potential oscillations in Hb9 interneurons was somewhat higher than typically used to evoke fictive locomotion (5–6 μM; Fig. 3). However, the lower NMDA concentration is normally added in the presence of 5-HT, which could reduce the minimal NMDA concentration needed to evoke oscillations. Ziskind-Conhaim et al. [49] present evidence that the TTX-sensitive persistent-sodium current (I_NaP_) is involved in generating, small amplitude voltage oscillations in synaptically isolated Hb9 interneurons. The authors propose different mechanisms for Hb9 bursting when induced by low (5 μM; I_NaP_ -dependent) and high (20 μM; I_NaP_ -independent) NMDA concentrations, and show that bursting evoked by high NMDA concentration is nickel-sensitive and may be due to nickel-block of NMDA receptors [50]. Our results are consistent with Ziskind-Conhaim et al. 's conclusions.

Although NMDA produced a concentration-dependent increase in both cycle frequency and voltage amplitude in Hb9 oscillations when spike-mediated synapses were blocked with TTX, these oscillations were not voltage-dependent (Fig. 4). Of the 7 neurons recorded, 5 produced small amplitude (<15 mV) and two produced large amplitude (>15 mV) oscillations in NMDA (21 μM) and TTX (1 μM). For most of these neurons (6 of 7), both voltage amplitude and cycle frequency of the NMDA-induced oscillations were voltage-independent (Fig. 4C). Previous work showed that the cycle frequency of large amplitude oscillations either increased [41] or decreased [62] with hyperpolarization of the membrane potential. Our experiments differ from these earlier studies in lacking dopamine and serotonin; either or both of these modulators could activate a cellular mechanism that produces the voltage-dependence for cycle frequency in Hb9 interneurons. Voltage independence of oscillations could also reflect the dependence of these oscillations on electrical coupling with other neurons (since fast synaptic interactions were blocked in TTX) [33], [54], [65]. Previous studies have provided firm evidence for electrical coupling of Hb9 interneurons to other neurons, though there was disagreement over whether Hb9 neurons are coupled to other Hb9 neurons [37] or to unidentified interneurons [7]. Our results suggest that the voltage-independence of the NMDA-induced oscillations reflects the strong electrical coupling of Hb9 neurons to other neurons (Hb9 or otherwise), and that this electrically coupled network is necessary to support rhythmic oscillations.

Voltage sensitive calcium currents play important roles in a variety of neuronal properties [38]. To determine whether NMDA potentiates I_Ca(T)_ in Hb9 interneurons, we applied a series of voltage-steps from −90 to −30 mV before and during NMDA (21 μM) application to the recording bath (Fig. 5). In an attempt to activate primarily I_Ca(T)_, we restricted the upper limit of the voltage step to −30 mV. It is likely, however, that high-threshold calcium currents were also weakly activated at −30 mV, since these currents have been detected in intersegmental commissural interneurons at voltages more depolarized than −40 mV [66], [67]. Based on this earlier work, the majority of the calcium current evoked by a voltage step from −90 to −30 mV (Fig. 5) is I_Ca(T)_, while the remaining current is probably due to the activation of high-threshold (P/Q or N type) calcium current. NMDA did not affect the amplitude of this calcium current, suggesting that it does not activate a signal transduction pathway that modulates I_Ca(T)_. It has been suggested that NMDA promotes voltage oscillations in other neurons through non-linear membrane characteristics, such as a shift of the Mg^2+^-dependent region of negative slope conductance in the NMDA I/V relationship [57], [58]. The data presented here do not provide any insight into this possibility.

Although Hb9 interneurons are rhythmically active during fictive locomotor-like behavior [40], [41], [47]–[50] and may play a role in generation of the locomotor pattern [45]–[47], [49], it is still not clear if the NMDA-induced Hb9 oscillations participate in the organization and generation of the locomotor pattern. Our present study does not address this question, except to point out that NMDA alone does not enhance I_Ca(T)_, a current expressed in Hb9 interneurons that is thought to contribute to rhythmogenesis [35]. Other neuroactive compounds, such as serotonin or dopamine, may modulate I_Ca(T)_ in Hb9 and other interneuron classes, to support the CPG generation of locomotor activity.
